# Dog Guardian Interpretation of Familiar Dog Aggression Questions in the C-BARQ: Do We Need to Redefine “Familiar”?

**DOI:** 10.3390/ani15192876

**Published:** 2025-09-30

**Authors:** Sarita Pellowe, Carolyn Walsh

**Affiliations:** 1 Cognitive and Behavioural Ecology Program, Memorial University of Newfoundland, St. John’s, NL A1C 5S7, Canada; info@eastcoastcanine.ca; 2 East Coast Canine Dog Training, St. John’s, NL A1E 3A5, Canada; 3 Department of Psychology, Memorial University of Newfoundland, St. John’s, NL A1B 3X9, Canada

**Keywords:** dog rivalry, canine behavior, FDA

## Abstract

**Simple Summary:**

The popular Canine Behavioral Assessment and Research Questionnaire (C-BARQ) contains a four-question subscale asking dog guardians to score their dog’s threatening behaviours towards another dog in the household, which generates a score for familiar dog aggression (FDA) or dog rivalry. In our C-BARQ scores from another study, we noticed a large number of participants who lived with only one dog (58.6%) completed these questions, giving their singleton dog an unexpected score. This could be a problem for understanding what these scores mean. We wondered if lifestyle factors might be related to guardians interpreting the meaning of “familiar dog” more broadly, so we sent them a follow-up questionnaire. Singleton dogs given an FDA score had proportionately more social activities with non-household dogs compared to singleton dogs with no score. We then reviewed the literature to see if other studies had similar findings for singleton dogs. We found that many studies did not report FDA scores for various reasons, and in those that did so, few provided information on the proportion of singleton dogs with FDA scores. We encourage researchers to consider ways to make the FDA scores more consistent and interpretable across studies so that we might better understand dog rivalry behaviour.

**Abstract:**

The C-BARQ familiar dog aggression (FDA) subscale contains four items relating to threatening responses towards familiar dogs in the same household (i.e., dog rivalry). In a recent study, we noticed that 92 of 157 guardians who owned only one dog completed the FDA items, generating an unexpected score. We followed up with participants to explore whether lifestyle factors influenced their completion of the FDA items. Singleton dogs with FDA scores were more likely to regularly participate in social activities with other dogs, with many scores based on such interactions with non-household dogs. The singleton dogs with FDA scores also had marginally lower fear-related C-BARQ scores compared to singletons with no FDA score and dogs living in multi-dog households. We then conducted a scoping review of articles using English versions of the C-BARQ and found wide variation in whether or not FDA scores were reported. Studies that reported significant FDA findings often did not indicate the proportion of scores in their data that came from singleton dogs, raising issues of accuracy and interpretation of the subscale. We discuss ways to clarify the interpretation of the FDA questions by dog guardians and hope to promote further consideration of practices to improve replicability across studies.

## 1. Introduction

The Canine Behavioral Assessment and Research Questionnaire (C-BARQ), introduced over 20 years ago [1], is a popular and useful questionnaire completed by dog owners/guardians to generate an inventory of dog behaviour (particularly problematic behaviour) by providing scores for 14 behaviour categories [2]. One of these categories or subscales is familiar dog aggression (FDA), also referred to as dog rivalry, defined as “threatening or hostile responses to other familiar dogs in the same household” [2,3]. To obtain an FDA subscale score, dog guardians/owners are asked to rate four items comprising different contexts in which their dog may show signs of aggression towards another dog, including the following: (1) “Towards another (familiar) dog in your household”; (2) “When approached at a favourite resting/sleeping place by another (familiar) household dog”; (3) “When approached while eating by another (familiar) household dog”; and (4) “When approached while playing with/chewing a favourite toy, bone, object, etc., by another (familiar) household dog” [2]. The rating scale ranges from “0—No visible signs of aggression” to “4—Snaps, bites, or attempts to bite”. While the term “familiar dog” is not explicitly defined in the C-BARQ, the wording in each question includes “another (familiar) household dog”, i.e., refers to another dog living in the same home. In a recent study we conducted [4], we used the C-BARQ as a means to investigate relationships between gut microbiota and behaviours in a companion dog population. Surprisingly, almost 60% (92/157) of respondents who lived with only one dog completed the questions for familiar dog aggression; therefore, their dog received an FDA subscale score that we describe as “unexpected”. That such a large proportion of participants with singleton dogs completed the FDA subscale items raises questions around how dog guardians interpret the FDA items, as well as around how the C-BARQ FDA score has been reported and interpreted in other published studies.

Several studies have reported that FDA scores in dogs are significantly related to other behavioural and lifestyle factors. These include correlations between a dog’s FDA score and both guardian personality and gender [5]. negative outcomes in puppy prison programs for dogs with higher FDA scores [6], increased FDA scores in dogs with atopic dermatitis [7], and decreased FDA scores in dogs from both hoarding situations [8] and commercial breeding establishments [9]. Familiar dog aggression is also reported to differ significantly between breeds [10,11,12,13,14]. However, if such findings are based on FDA scores that have included singleton dogs, then their validity and interpretation are unclear.

Some studies have acknowledged the inherent environmental requirement that a dog live with at least one conspecific to receive the FDA score, and they note that these questions would be irrelevant for dogs living alone, resulting in no FDA scores for singleton dogs (e.g., [14,15,16]). Additionally, since all C-BARQ subscale scores are presented as an average of multiple questions (four in the case of the FDA subscale), some studies have defined a threshold for missing values in their subscale calculations; for example, Duffy and Serpell stated that if more than 20% of the questions comprising any subscale were not answered, then the mean subscale score was not calculated [17]. However, this may not fully account for guardians of singleton dogs who possibly answered at least three of the four FDA-related questions. Despite not completely addressing the assignment of FDA scores to singleton dogs, implementing such a screening process for missing values can increase the reliability and validity of all subscale scores. Similarly, Broseghini and colleagues translated the English C-BARQ into Italian and completely removed the dog rivalry/FDA subscale from further analysis, as the four subscale items had missing data rates of 25% or higher [18]. While we agree that this is sound data management, it is unfortunate that the process likely expunged “legitimate” FDA scores for dogs living in multi-dog homes, and dog rivalry could not be evaluated further. The C-BARQ instrument (rightly) provides researchers with all responses from study participants and calculates scores for subscales with missing values. Thus, researchers must manually screen for missing values. In addition, there is no screening question for the eligibility of dogs to receive the FDA score in the C-BARQ itself, aside from instructions to select N/A if the question or context is irrelevant to the participant.

Given the proportion of singleton dog guardians in our study who responded to the FDA questions, we decided to evaluate how common this practice might be, particularly in light of the popularity and obvious usefulness of the C-BARQ tool in canine science (i.e., at the time of this writing, 132 papers are listed on the C-BARQ website [19]). If studies have reported and evaluated CBARQ’s FDA scores for singleton dogs who do not live with other dogs in the household, further consideration of how FDA results are interpreted may be warranted.

How do dog guardians interpret the FDA questions when they complete the C-BARQ? Specifically, why do some guardians of singleton dogs answer the questions, apparently ignoring the text referring to “another familiar (household) dog”? One obvious possibility is that they may be referring to another household dog who no longer lives with their current dog, perhaps because of rehoming or the death of the other dog(s). Another possibility is that they respond to the FDA items using their dog’s regular interactions with familiar dogs with whom they do not live. Interestingly, these responses allow us to compare whether the (currently) singleton dogs of guardians who do respond to the FDA items have had different experiences with other dogs compared to the singleton dogs of guardians who do not respond to the FDA items. Such different experiences may predispose the guardians to interpret the FDA questions more broadly. Additionally, we wondered if guardians of singleton dogs with an unexpected FDA score also scored their dogs differently for other C-BARQ sub-scales, perhaps because either the dogs are behaviourally different or the guardians are interpreting the C-BARQ questions in a different manner (e.g., over- or under-reporting behaviours).

This current research report consists of two studies. In Study 1, we asked participants from our original study [4] to complete a follow-up questionnaire so we might better understand their FDA responses. We explored whether there were differences in C-BARQ subscale scores between the dogs of guardians who responded as expected to the FDA questions (i.e., dogs either received a score if in a multi-dog home, or no score if they were singleton dogs) compared to guardians who interpreted the FDA questions differently (i.e., those with single-dog homes who completed the FDA questions). We also explored their historical living arrangements with other dogs, as well as some social factors that may impact dogs’ familiarity with other dogs who do not live in their household, such as the frequency and location of social activities with other dogs. We expected that singleton dogs receiving FDA scores would likely have lived with other household dogs in the past and/or would be more likely to socialize with non-household dogs. We also expected that the FDA scores in the current study would increase for the same dogs compared to the scores obtained in the original study, not necessarily because of an increase in dog rivalry behaviour but due to the FDA-focussed nature of the questionnaire introducing demand characteristics in which participant responses are altered because they are aware of the study’s intent (e.g., [20,21]). In Study 2, we conducted a scoping review that explored the extent to which studies using the C-BARQ may have reported FDA scores for singleton dogs. We wished to establish if our finding was a result of our study design or if it reflected a wider pattern in studies reporting C-BARQ outcomes. We expected that our study participants were not unique in their interpretation of the FDA items and that studies either excluded singleton dog FDA scores via a screening process, or they may not have employed a screening process and, therefore, perhaps inadvertently reported FDA scores for dogs living with no other household dogs. In obtaining this information, it is not our intention to criticize specific studies or the C-BARQ tool itself, which has generated a remarkable amount of information about pet/companion dog behaviour over the past two decades. Rather, we wish to encourage a broader discussion and investigation about the meaning of FDA (dog rivalry) subscale scores and to begin a conversation around ways to provide more clarity for dog guardians who are completing the C-BARQ, as well as for researchers who are evaluating the relationship of familiar dog aggression with other factors of interest. As the use of guardian-based questionnaires in canine science is not limited to the C-BARQ, such a conversation may encourage the broader evaluation of how guardians interpret terminology in other instruments.

## 2. Materials and Methods

**Study 1—Follow-up FDA Questionnaires**

Dog guardians who had previously completed both the Diet and Lifestyle Questionnaire (Supplementary Materials S1) and C-BARQ between 6 May and 5 July 2021 were contacted via email and invited to participate in a follow-up questionnaire (Table 1) to investigate how they interpreted the FDA section of the C-BARQ. The follow-up questionnaire in this study, delivered via Qualtrics [22], was available between 4 May and 16 June 2022 and comprised 14 questions relating to the dog’s current living arrangements, social activities, and the 4 FDA questions from C-BARQ (Supplementary Materials S2). Of particular interest was the dogs’ experiences of socializing with other dogs; two questions addressing this were (Q9) “*Do you take your dog to visit other dogs at their home, or have other dogs over to visit at your home? If so, how frequently does this occur? These ‘other dogs’ might belong to neighbours, family or friends.*” and (Q10) “*Do you take your dog to socialize with other dogs away from a household environment (*e.g., *group walks/hikes with other dog guardians, dog parks)? If so, how frequently does this occur?*”.

Participant responses were grouped based on living arrangements, i.e., target dogs who lived with conspecific(s) or who did not, and the FDA scores were then categorized by their relevance to these living arrangements (i.e., the dog received either an expected or unexpected FDA score). Dogs were then further assessed by their dog cohabitation history (i.e., either had, or had not, previously lived with other dogs) and frequency of socialization with other dogs (both on and off their property). At the end of the questionnaire, participants were specifically asked which dog they were thinking about if/when they answered the FDA questions to better understand their interpretation of the term “familiar dog”.

### Statistical Analysis

As the data in this study were non-normally distributed, non-parametric tests were selected for the statistical analyses. C-BARQ subscale scores collected in the original study prior to follow-up were analyzed based on the FDA outcome (i.e., expected vs. unexpected score), and tested for significance using the Mann–Whitney U test. Further analyses used Kruskal–Wallis and Dwass–Steel–Critchlow–Fligner pairwise comparisons between single dogs with expected FDA scores, single dogs with unexpected FDA scores, and multi-dog-household dogs with expected FDA scores. These analyses explored 9 C-BARQ subscales; thus, *p*-values were considered significant below 0.0055 after Bonferroni correction [23] and marginally significant below 0.05. Social activity, both in and out of the home, was investigated for each group using the chi-square test for association with Fisher’s exact test. Statistical analyses were completed in Jamovi (version 1.6.23.0) [24].

**Study 2—Scoping Review**

This scoping review covered studies published between 2003 (when C-BARQ was first validated) and 1 August 2023 (the date of our most recent search for the current review). While the C-BARQ has been validated in multiple languages (e.g., [18,25,26,27]), we limited papers in the review to those in which the familiar dog aggression questions were presented in the English language, as translations into other languages may have differing effects on the interpretation of the questions presented.

Journal databases compatible with Covidence [28] and accessible via Memorial University Library were searched using the following terms:

{C-BARQ} OR {CBARQ} OR {C BARQ} OR {Canine Behavioral Assessment and Research Questionnaire} OR {Canine Behavioral Assessment and Research Questionnaire} AND PUBYEAR > 2002 AND PUBYEAR < 2024

AND (LIMIT-TO (LANGUAGE, “English”))

The journal databases utilized in the literature searches were Web of Science, PubMed, CINAHL, Embase, MEDLINE, PsycInfo, Scopus, Google Scholar, and World Health Organization (WHO). Each database search was uploaded into Covidence in .RIS (PsycINFO, CINAHL, Embase, Scholar, Web of Science, Scopus, MedLINE) or .txt (PubMED) format. During the initial abstract screening, papers were included based on the mention of C-BARQ in the abstract or title. Papers with ambiguous methodological descriptions in the abstract, for example “used a guardian-reported survey”, were tentatively included to be further investigated during the full text review. Papers were excluded at the abstract screening stage if they clearly did not use C-BARQ, if they were not primary research (i.e., review papers, theses, textbooks, presentation or conference summaries were excluded), or if either the published paper or version of C-BARQ used was not delivered in English. Once the abstracts were screened by two reviewers, they proceeded to the full text review, during which papers were more closely investigated. Each reviewer voted to include or exclude each paper. In the event of a disagreement between reviewers, the full text was reviewed together to confirm eligibility criteria, which required that the article be a peer-reviewed empirical study written in English, in which the C-BARQ was presented to study participants in English.

Upon compiling the final list of eligible articles, we assessed the reporting of scores in each study, recording the version of C-BARQ reported—full, mini (42 question), or custom/specifically-modified—and whether FDA scores were reported. For studies reporting FDA results, we then tallied if the FDA scores were significantly related to other factors investigated in the study. We also charted if the studies had excluded FDA data and if they provided justification for doing so. We recorded whether each study included a complete definition of FDA and whether there was an overt criterion for reporting any subscale scores when there were missing values within the subscale questions. Our results provide a tally of studies with each of these characteristics. Finally, any available open access quantitative datasets for the final list of eligible articles were downloaded so that we could evaluate whether the number of dogs living in a household was included as a question and, if so, calculate the proportion of singleton dogs who received an FDA score.

## 3. Results

### 3.1. Study 1—Follow-Up FDA Questionnaires with Local Participants

Of the 235 respondents to our original C-BARQ and Diet and Lifestyle questionnaires in 2021, 76 participants were successfully recruited one year later (2022) for the follow-up study. At the time the follow-up questionnaire was delivered, 51 dogs were living as singleton dogs, while 25 were living in multi-dog households. When compared to the original questionnaire data, a total of five dogs had changed living arrangements; one dog had been living with another dog in 2021 but was now a singleton, while four dogs who were living alone in 2021 now had another dog in the home. These five dogs were excluded from the analysis due to the potential impacts of the changes in living arrangements on their interpretation of “familiar dog” between the original and follow-up questionnaires. This left 50 single-dog and 21 multi-dog households in the study (final *n* = 71 dogs).

#### 3.1.1. Behavioural Profiles Differ in Singleton Dogs with FDA Scores

The portion of the original cohort (2021, *n* = 235) that received an FDA score (*n* = 170) was compared to those in the follow-up cohort (2022, *n* = 71), based on their 2021 (*n* = 46) and 2022 (*n* = 57) responses to the FDA questions (Table 2). For those dogs who received an FDA score in both the original and follow-up studies (*n* = 42), the mean score increased significantly from 0.311 ± 0.08 to 0.649 ± 0.12 (W = 381, *p* < 0.001, Wilcoxon), and the median score increased from 0 to 0.5.

Of the 50 singleton dogs that were reported on in the follow-up survey, 25/50 had received an FDA score in the original 2021 questionnaire. The remaining 21 (of the total 71) dogs in 2022 were living in multi-dog homes, and all had received an FDA score (as expected) in the original 2021 study. Based on this information, dogs were assigned to one of the following three groups: singleton dogs with an FDA score (Group 1, *n* = 25), singleton dogs with no FDA score (Group 2, *n* = 25), and dogs from multi-dog homes with an FDA score (Group 3, *n* = 21). There were no dogs from multi-dog homes who did not receive an FDA score.

**Figure 1:**
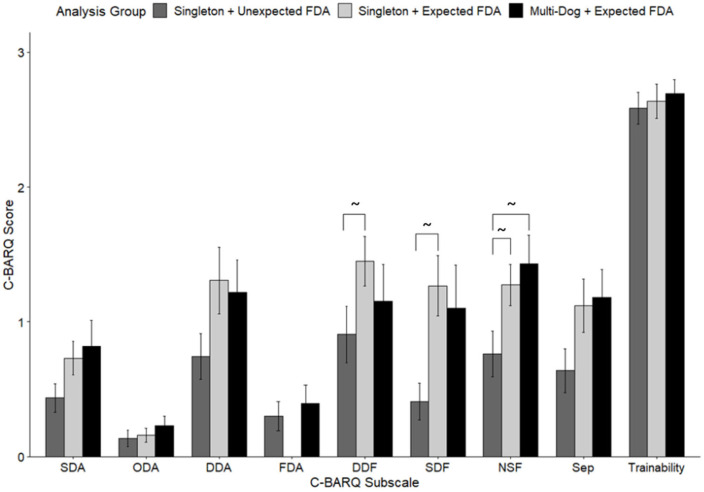
Comparison of C-BARQ subscale scores from 2021 for dogs who lived alone and received an FDA score (Group 1—Unexpected FDA, dark grey, *n* = 25), dogs who lived alone and did not receive an FDA score (Group 2—Expected FDA, light grey, *n* = 24), and dogs who lived in multi-dog homes and received an FDA score (Group 3—Expected FDA, black, *n* = 21). C-BARQ subscales included stranger-directed aggression (SDA), guardian-directed aggression (ODA), dog-directed aggression (DDA), familiar dog aggression (FDA), dog-directed fear (DDF), stranger-directed fear (SDF), non-social fear (NSF), separation-related issues (Sep), and trainability. Marginally significant differences (0.0055 < *p* < 0.05; denoted with ~ between groups) were seen between Groups 1 and 2 for dog-directed fear (DDF, *p* = 0.037), stranger-directed fear (SDF, *p* = 0.008), and non-social fear (NSF, *p* = 0.032). NSF was also marginally different between Groups 1 and 3 (*p* = 0.027) (Kruskal–Wallis with DSCF pairwise comparisons, after Bonferroni corrections for multiple comparisons *p* < 0.0055 was considered significant).

Pairwise comparisons of C-BARQ subscale scores indicated that the majority of significant differences were between singleton dogs with an unexpected FDA score (Group 1) and singleton dogs without an FDA score (Group 2; Figure 1). Compared to these singleton dogs without an FDA score, Group 1 dogs displayed marginally lower dog-directed fear (DDF; 0.907 ± 0.21 vs. 1.45 ± 0.18, W = 3.479, *p* = 0.037), lower stranger-directed fear (SDF; 0.410 ± 0.14 vs. 1.27 ± 0.23, W = 4.21, *p* = 0.008), and lower non-social fear (NSF) scores; 0.762 ± 0.17 vs. 1.27 ± 0.15, W = 3.564, *p* = 0.032). Only one marginally significant difference was noted between singleton dogs with an FDA score (Group 1) and dogs from multi-dog homes (Group 3), which was that the NSF subscale scores were lower for the former (Group 1, 0.762 ± 0.17 vs. Group 3, 1.39 ± 0.21; W = 3.646, *p* = 0.027). No significant differences were seen between Group 2 (singletons with no FDA score) and Group 3 dogs.

#### 3.1.2. Reported Socialization Behaviour

Proportionately fewer singleton dogs with an FDA score (Group 1) were reported by their guardians to never experience in-home social visits (12%), compared to dogs in Groups 2 (36%) and 3 (19.1%), while proportionately more Group 1 dogs experienced these in-home visits greater than once per week (32% vs. 4.0% and 4.7%, respectively; Table 3, *p* = 0.026, Fisher’s exact test). A similar pattern was seen for social visits that occurred away from a home (e.g., outdoors; Table 3, *p* = 0.041, Fisher’s exact test).

Singleton dogs with an unexpected FDA score were further separated based on their historical living arrangements; either they had lived with other dogs (Group 1A, *n* = 11), or they had not (Group 1B, *n* = 14). These two subgroups were compared to singleton dogs with no FDA (Group 2) and dogs from multi-dog homes with FDA scores (Group 3). There were no significant differences between C-BARQ subscale scores for Groups 1A and 1B. The only marginally significant finding was a lower stranger-directed fear (SDF) score for Group 1A (singleton dogs with FDA score, had lived with other dogs previously) compared to Group 2 (singleton dogs with no FDA score; 0.364 ± 0.25 vs. 1.27 ± 0.23 respectively, W = 3.721, *p* = 0.042, Kruskal–Wallis with Dwass–Steel–Critchlow–Fligner pairwise comparisons). When all dogs were based solely on their historical living arrangements (i.e., regardless of current group assignment, they had or had not lived with other dogs), there were no significant differences in C-BARQ scores.

#### 3.1.3. Guardian Reasoning for Completing FDA Items

Guardians were asked specifically which dog they had been referring to when they answered the familiar dog aggression questions (Table 4). Participants with an unexpected FDA score and singleton dogs who had previously lived with other dogs but currently did not (*n* = 11) showed the most diversity in their responses; four (44%) guardians selected a dog they previously owned, three (33%) referred to a friend or family member’s dog spending time in their home, two (22%) referred to a dog outside the home, and two (22%) provided no response. Participants with singleton dogs who had received an unexpected FDA score, but had not previously lived with other dogs (*n* = 14), mostly selected responses relating to socialization, such as “A friend or family member’s dog who spends time in your home” (nine participants; 64%) or “A friend or family member’s dog who spends time away from your home” (3 participants, 21%), with two participants not responding. Participants with singleton dogs who did not originally receive an FDA score (*n* = 25) still had the opportunity to provide an interpretation of “familiar dog”; six (24%) referred to a friend or family member’s dog spending time in their home, and eight (32%) referred to a dog outside the home, while four participants (16%) provided no response. This was the only group to utilize the “Other” selection (seven participants, 28%), citing “N/A” (four participants), a previous roommate’s dog (one), a dog they met regularly (one), and only one dog in the house (one). As expected, all participants with multi-dog homes selected “Another dog that you own” (21 participants).

### 3.2. Study 2—Scoping Review

**Figure 2:**
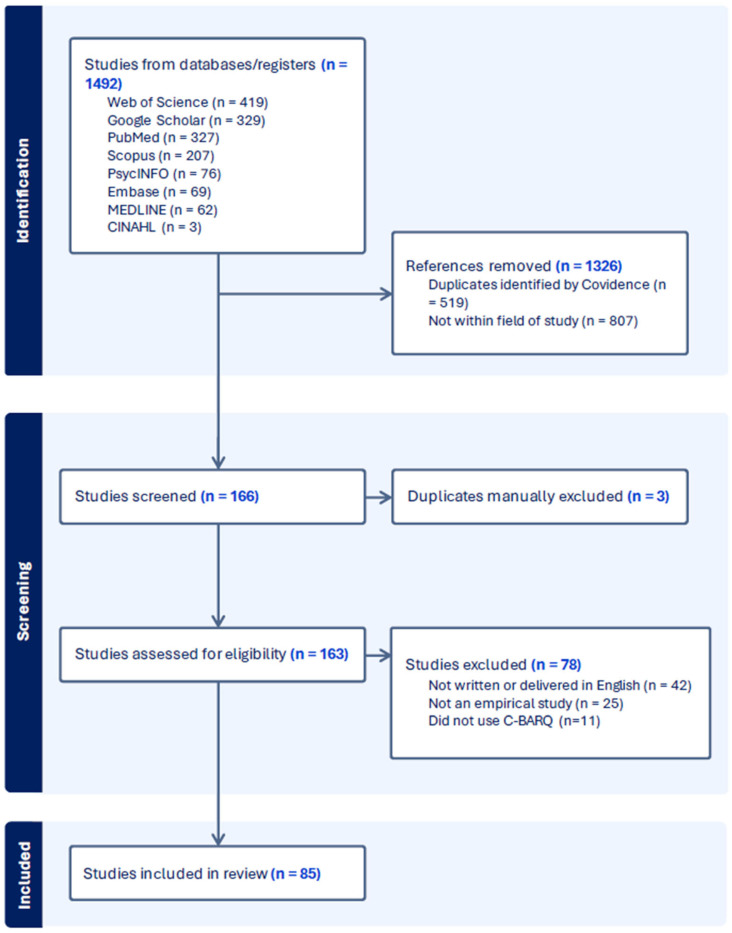
PRISMA-ScR flowchart for studies screened by Covidence and assessed manually by two independent reviewers for eligibility criteria. The final search was conducted on 1 August 2023.

The most recent literature search was conducted on 1 August 2023 and returned a total of 1492 papers for processing through Covidence (Figure 2). Covidence identified 519 duplicates, and a further 807 papers were excluded, as they were not within the field of study. We identified 166 papers that were potentially eligible for inclusion, of which 81 were manually removed; three were duplicates; 42 were not written or delivered in English; 11 did not use C-BARQ; and 25 were not empirical studies. After manual screening, 85 papers were identified for the analysis (listed in Supplementary Materials S3).

As shown in Table 5, 63 of 85 papers reported using the full version of C-BARQ; 22 papers reported the C-BARQ contained 100 questions, 17 papers reported 101 questions, 16 papers did not report the number of questions in their version of C-BARQ, two papers reported C-BARQ to have 102 questions, one reported 103 questions, and one reported 105 questions. The original C-BARQ paper was also included, which reported an original 152 questions prior to validation. Not all papers in the review used the full version of C-BARQ; 11 papers used the mini C-BARQ consisting of 42 questions (originally evaluated in [29]), and 11 papers either extracted individual subscale questions relevant to their study from C-BARQ or did not report sufficient detail to confirm the format of C-BARQ used in their study.

For studies that used the full C-BARQ, 37/63 reported on the FDA subscale, of which 18 indicated significant findings related to FDA scores (references in Table 5 footnote). Some studies using the mini C-BARQ also included the FDA; 7/11 papers reported FDA scores, of which four were significantly related to other factors in the study. Finally, of the 11 papers that utilized a modified test (or did not provide sufficient information for us to determine which C-BARQ version they used), only one reported a significant finding related to the FDA subscale score.

#### 3.2.1. Definition of Familiar Dog Aggression

A total of 37 studies that used the full version of C-BARQ included results for FDA; however, a definition of the FDA subscale was provided in only 14/37 studies. Of the 18 papers that reported a significant result for FDA, eight provided a clear definition of the subscale [6,7,11,12,14,31,35,36]. Of the seven studies that used the mini C-BARQ and reported FDA scores, none provided a clear definition for the subscale; however, four of these papers reported significant findings related to the FDA. Finally, of the papers that used a modified C-BARQ or did not provide enough information to determine the form used, one paper clearly defined the subscale and was also the only paper to report significant findings [40].

#### 3.2.2. Missing Responses in C-BARQ Subscales

Based on the broad scope of the C-BARQ, not all questions would be relevant to every participant; for example, some dogs may not have experience within the context of a specific question, and therefore, guardians cannot report on a behaviour they have not observed. This inclusion criterion is sometimes reported in the study methods and is limited neither to the FDA subscale nor to any version of the C-BARQ. Missing values were reported by some authors as a justification for the removal of subscales from further analyses; 32% (27/85) of articles reported screening criteria for removing scores with missing values, while 68% (58/85) mentioned neither screening for missing values nor an exclusion threshold for missing values.

Almost half of the 85 studies (41) did not report FDA scores in their results; most (25/41) of these articles did not provide a justification for the omission of FDA from their results, and the remaining articles (16) primarily stated either a threshold requirement for inclusion based on missing values (i.e., there were too many missing values to calculate the subscale score) or that aggression subscales were beyond the scope of the study.

#### 3.2.3. Quantitative Data Available for FDA

Articles that included supplementary materials or quantitative data were assessed to identify whether it was likely that some of the dogs receiving FDA scores were singleton dogs. Only four papers provided supplementary materials that included open-source data relevant to FDA [13,38,41,42]. While three papers provided information regarding the number of dogs with FDA scores in the supplementary materials [38,41,42], they did not provide information on the number of dogs living with conspecifics in the home in the text of the paper, and they did not report any significant FDA findings. Only one of the previous four studies [13] reported a correlation with FDA scores (competitive dog status) and also reported information in the supplementary files regarding whether dogs lived as singletons or not. In that study, of 397 dogs, 329 dogs received an FDA score, with the dataset indicating that there were 150 dogs reported as singleton dogs (i.e., 247 living with other dogs) [13]. Based on this information, and assuming that all dogs living in multi-dog homes received an FDA score, we calculated that up to 82/150 (54.6%) of singleton dogs were given an FDA score, which is similar in magnitude to our own finding (58.5%).

While other studies did not provide data in the supplementary materials (and we did not seek the data from the authors), information regarding missing values for FDA scores [6,10,43] and percentage of dogs in singleton/multi-dog homes [30] could be found in the results and/or discussion sections. These four studies all report that data were missing for the FDA subscale, although relative proportions of FDA scores that might have been assigned to singleton dogs cannot be determined. In an additional study [36], the authors acknowledged a discrepancy between the number of dogs receiving an FDA score and the number living alone; they reported that 85% of the dogs received a score for FDA and noted that 191 respondents (37%) were reporting on single-dog homes (i.e., 63% of the sample consisted of dogs from multi-dog homes). These percentages indicate that some singleton dogs in the study would have received a score for FDA; for example, if 63% of respondents were from multi-dog homes, and 85% of all respondents received an FDA score, there is the potential for 12% of the dogs with FDA scores to be singletons.

## 4. Discussion

Based on our questionnaire outcomes and the scoping review, it appears that the questions comprising the familiar dog aggression (FDA) subscale in the popular C-BARQ are often answered by guardians of singleton dogs, even though the wording of items in the subscale clearly indicates its intent to evaluate aggression towards other dogs who live in the same household. This might not be a major concern, if most guardians are responding to the FDA items on the basis of their dog’s past behaviours towards a previously cohabiting/household dog. However, our follow-up questioning with a small sample of guardians of singleton dogs with unexpected FDA scores suggests this is rarely the case; most had never lived with another dog, and of those who had, some guardians reported that they answered the FDA items while thinking about their dog’s behaviour towards a familiar but non-cohabiting/non-household dog. Interestingly, in our sample, the singleton dogs with FDA scores had relatively higher levels of social interaction with other (familiar, non-household) dogs compared to both singleton dogs without FDA scores and dogs from multi-dog homes. Moreover, scores for some other C-BARQ subscale scores suggested further behavioural differences between groups; dog-directed fear (DDF), stranger-directed fear (SDF), and non-social fear (NSF) were all marginally lower in singleton dogs with FDA scores than in singletons with no FDA scores. Singleton dogs given FDA scores also had marginally lower NSF scores than did dogs from multi-dog homes.

These unexpected FDA scores for singleton dogs may have information of value and interest, both for guardians and for researchers. However, based on the items in the C-BARQ FDA subscale as currently written, we cannot ascertain the true proportion of singleton dogs who show rivalry behaviours towards familiar but non-cohabiting dogs, as we cannot know the proportion of guardians of such dogs who interpret the FDA items as written and, therefore, do not complete them because they live with only one dog, resulting in no FDA score for that dog. More concerning is the mixing of the FDA scores from singleton dogs and dogs living with other dogs in the household; the validity and interpretation of these scores, and those of any analyses relating such “mixed” FDA subscale scores to other factors, called into question, as the FDA scores do not clearly represent a single construct of aggressive behaviour towards a cohabiting dog, particularly with respect to resources that the dogs share and/or may compete for within the household.

Indeed, the four questions associated with the FDA subscale ask guardians to rank their dog’s general aggression towards another familiar (household) dog, when approached by the dog at a favourite resting place, while eating, and while chewing a toy/bone. Currently, there is no strict agreement on the definitions for terms related to this display of aggression within the clinical and consulting animal behaviour field, but “possession aggression” and “resource guarding” are commonly used (e.g., [44]). Typically, resource guarding is a behavioural issue that is problematic when dogs are living together and competing for access to the same resources (e.g., [45]). However, such resource-guarding behaviours can be exhibited towards any dog or person, whether they live with the dog or not, if the appropriate context is present. The extent to which the object of the aggression (i.e., household dog vs. an occasionally visiting dog in the home) may reflect possible differences in underlying motivations, if any, is unclear. Indeed, aggression towards non-cohabiting dogs entering a dog’s home could be related to territorial defence (e.g., [46]). Certainly, FDA scores for a dog that cohabits with another dog may reflect a combination of the dog’s temperament/personality, the nature of the relationship between the dogs, and/or the guardian’s management strategies for their dogs’ behaviours. As previous studies have suggested, living with conspecifics increases the opportunity for practicing resource-based aggression [45,47], thereby potentially increasing FDA scores for dogs in multi-dog homes compared to those for singleton dogs with occasional visitors. The importance of opportunity to express behaviour is also suggested in our data on singleton dogs with FDA scores; those dogs for whom their guardians completed the FDA items had proportionately more frequent in-home and out-of-home socialization experiences than did singletons without FDA scores. However, in our current sample, there were no significant differences between the average FDA scores of dogs who lived with other dogs (Group 3) compared to the average FDA scores of singleton dogs who received a score (Group 1). This suggests that the FDA score may reflect some threshold of opportunity for a dog to express rivalry behaviour; for example, exposure to other dogs is required for a dog to be assigned an FDA score, but above some minimum level, the frequency of such exposures may not impact the score further.

The extent to which guardians’ interpretations of dog behaviour influence their scoring of questionnaires on behaviour and dog personality may vary depending on the specific questions/instrument completed (e.g., [34]). However, there is evidence that guardian-reported behaviour corroborates with independent assessments of dog behaviour (e.g., [48,49]). It is possible that guardians of singleton dogs who experience frequent social interactions with other (non-cohabiting) dogs (and who complete the C-BARQ FDA items) may be more conservative about what they are willing to call a behaviour problem vs. a normal interaction between dogs, given that their dogs probably appear to them to enjoy interactions with their familiar conspecifics (as, otherwise, they likely would not continue). Interestingly, the non-FDA C-BARQ subscales on which these singleton dogs with FDA scores showed more favourable scores compared to singletons whose owners did not complete the FDA items were all fear-related. Given the differences in socialization experiences between the two groups of dogs, these fear-related differences in behaviour may reflect true differences between the groups, either because dogs who are fearful are less likely to be exposed to contexts in which other dogs and strangers are present and/or exposure to such contexts reduces the expression of fearful behaviours (although most research has focussed on the socialization experiences of puppies/young dogs, e.g., [50], reviewed in [51]). Given the relatively small sample size of the groups compared in this study, it would be worthwhile to evaluate C-BARQ scores for singleton dogs who experience different levels of social interaction as adults and compare them to dogs from multidog homes across larger datasets (e.g., using approach of [52]). The inclusion of these singleton dogs, who may have enhanced socialization experiences with other dogs (and people), may affect not only the FDA subscale score evaluation but potentially the relationship of FDA scores with other C-BARQ subscale scores, particularly those related to fear.

Our scoping review highlighted some inconsistencies in the reporting of C-BARQ data, particularly with respect to the reporting of FDA scores. It is almost certain that some portion of studies reporting FDA scores have included singleton dogs in their results, and most studies do not indicate whether or not they removed or included singleton dog scores for the FDA. Indeed, many appear to have not collected the additional data required to do so. In fact, no studies reporting FDA data that we evaluated in the scoping review acknowledged how the interpretation/inclusion of FDA/dog rivalry scores from singleton dogs may (or may not) be problematic. It was more likely for authors to acknowledge that removing FDA scores or entire dogs due to missing data in the FDA scale could be an issue in their data interpretation. For example, Flint and colleagues [52] stated that the removal of dogs based on missing values for FDA may have skewed their results, and they acknowledged the potential bias that may have been introduced in other subscales by removing these individuals. It is possible that other researchers using the C-BARQ are aware of the lack of consistency in how dog guardians respond to the FDA items, but there is not yet an agreed-upon method to redress this issue, leaving the reporting of FDA results to vary widely.

As a question about the presence of conspecifics living in the home is not included in the C-BARQ, researchers must use supplementary questionnaires to obtain any demographic or environmental factors that may be relevant to their research questions. In our scoping review, we found that only one of the four studies with open-access datasets had obtained information on the number of dogs living in the home [13]. Of 397 dogs in the study [13], 329 received an FDA score in the study. However, as there were 247 dogs from multidog homes (i.e., 150 singleton dogs), 82 of the dogs receiving FDA scores were singletons. This discrepancy is not acknowledged or reported in the article itself, which, admittedly, was primarily focussed on the association of genetic markers with problem behaviour. However, this example is consistent with how FDA results are typically reported in the literature.

In our current study, when the four questions comprising the FDA subscale were delivered out of the context of the entire C-BARQ and framed as a follow-up study specifically addressing familiar dog aggression, the mean FDA scores for these same dogs increased from the score provided one year prior. While the severity of the dogs’ aggression may have increased in the 12 months between the questionnaires, it is more likely that the participants were simply more sensitive to the study question due to demand characteristics [20,21]. Although the score increase was not a concern for our specific research question here, it is worth considering that subscale items when presented in isolation from the entire C-BARQ instrument may generate scores that are inflated compared to the subscale scores obtained when participants are asked to complete all questions.

So, is it a fundamental problem that the interpretation by many dog guardians of the C-BARQ FDA items involves a broader definition of “familiar” that includes non-cohabiting/non-household dogs? From a psychometric testing perspective, this subscale may lack validity, the meaning of which has generated debate among researchers (see [53]), but it can be defined very basically as “the extent to which an assessment method measures what it is intended to” [54]. As the FDA items instruct guardians to respond on the basis of their dog’s behaviour to a “familiar (household) dog”, and many clearly do not limit their responses in this way, at the very least, accuracy and precision are lacking in the subscale. Does this mean, however, that the responses obtained from guardians about their singleton dog’s aggression directed towards familiar dogs with whom they do *not* live is uninformative? No, on the contrary, we feel that such data can be valuable and should be collected, evaluated, and interpreted, although with the following major caveat: FDA scores for dogs who live alone should be evaluated separately from the FDA scores for dogs who live with other dogs, at least until such a time it is clear whether or not the reported behaviours reflect the same underlying causes and motivational processes. This would allow researchers to evaluate dog rivalry behaviours among housemates who share access to resources and are managed by the same guardian separately from those behaviours shown among familiar dogs who do not share resources or necessarily experience the same management practices. In practice, this recommendation could involve expanding the current definition of “familiar dog” in the C-BARQ to include non-household dogs, or other possible modifications to the instrument itself. Alternatively, researchers using the C-BARQ could be encouraged to pose additional questions to confirm the number of dogs in the home, as well as, perhaps, some measure of the frequency of socialization with other (non-cohabiting) dogs. Certainly, how the FDA results are reported could become more consistent, with clear information provided in the results sections of articles on the proportion of respondents reporting FDA scores for dogs living with other dogs vs. dogs living as singletons. If there are no significant differences between these groups of dogs on other C-BARQ measures, it may be defensible to combine the groups for further analyses of FDA, or there may be more value in analyzing the groups separately, depending on the objectives of the research. While we do not claim to have definitive answers with respect to the best practices for using and interpreting the C-BARQ FDA subscale moving forward, we believe that a broader discussion about what these best practices could be is worthwhile and would likely improve consistency and reproducibility between studies.

## 5. Conclusions

Our work has shown that the interpretation of the familiar dog aggression (FDA) questions in the C-BARQ differs between dog guardians and is most likely influenced by their participation in social activities with other dogs that do not belong to the household. The relatively high rate of responses to the FDA questions by guardians of singleton dogs indicates that displays of aggressive behaviour towards familiar dogs (dog rivalry) are not limited solely to dogs who live in a multi-dog home, as dogs who frequently socialize with others may have adequate opportunity to experience the contexts queried by the FDA items. Although the completion of the FDA items by guardians of singleton dogs technically decreases the validity of the subscale, any removal of such dogs’ data, based on the assumption that they do not experience the appropriate contexts for dog rivalry, seems inappropriate as it excludes singleton dogs who have opportunities to display resource-based aggression. Instead, further research should investigate whether resource-based aggression differs when the target is a family dog, or an occasional visitor, and if the effects of management and learning history on the severity of these behaviours differ when the context is experienced daily (for multi-dog homes) or occasionally (in the case of singletons with visitors).

It is important that researchers are aware of the challenges presented by the FDA subscale when designing a behavioural study and that adequate lifestyle information is collected from dog guardians in order to better understand their responses to the subscale. We propose that the FDA items in the C-BARQ could benefit from more precise wording, or an updated definition of “familiar”. This change could be either *less* open to broad interpretation by guardians, i.e., familiar dog aggression as a threatening or hostile response towards “another dog that you own and who lives in the same home”, or instead, *more* open to wider inclusion of other non-cohabiting dogs, i.e., “a dog that your dog regularly spends time with your dog in your home or elsewhere”. The risk of the first suggestion is that guardians will continue to ignore or misinterpret the instructions, and some proportion will provide responses based on their dog’s behaviours towards other dogs with whom they do not live. Additionally, changing the FDA wording/definition alone, as in the second suggestion, would not be sufficient to separate the dogs who live with other dogs from those who live alone and socialize frequently, and it would not address any potentially different motivations for the rivalry behaviour (or other behavioural differences) between the two groups. To address this, we encourage researchers to consider adding additional questionnaires to accompany the C-BARQ in order to confirm other factors that are not specifically addressed in C-BARQ itself (i.e., the presence of conspecifics, frequency of socialization). Of course, these questions could be integrated into a revised version of the C-BARQ FDA subscale, with any “unexpected” FDA scores for singleton dogs flagged for researchers. While there are multiple ways to address the inconsistencies in how dog guardians report on their dog’s familiar dog aggression using the C-BARQ FDA subscale, we encourage researchers using this valuable instrument to be sensitive to the issue, and to be as transparent and as thorough as possible with their reporting of any additional screening and exclusion processes they employ in data analysis. This will undoubtedly improve reporting practices and reproducibility among studies and enhance our understanding of familiar dog aggression/dog rivalry behaviours.

## Figures and Tables

**Table 1:** Follow-up questionnaire delivered via Qualtrics between 4 May and 16 June 2022.

Question Number	Question	Response Options
Q1–4	Participant name, contact information, dog’s name and breed	
Q5	How many dogs currently live in your home?	1, 2, 3, 4+
Q6	How long has your household had this number of dogs?	Less than 1 month, 1–3 months, 3–6, 6–12, over 12 months
Q7	If your dog currently lives alone, have they previously lived with another dog?	Yes, No, N/A
Q8	If yes, which of the following statements best describes the transition from living with another dog to becoming the only dog in the household (Select multiple):	The other dog passed away; The other dog belonged to a friend/family member and living arrangements changed; The other dog was rehomed; The other dog was a temporary foster; Not applicable; Other
Q9	Do you take your dog to visit other dogs at their home, or have other dogs over to visit at your home? If so, how frequently does this occur? These “other dogs” might belong to neighbours, family, or friends.	Never; Once a week or less; More than once a week; Every day
Q10	Do you take your dog to socialize with other dogs away from a household environment (e.g., group walks/hikes with other dog owners, dog parks)? If so, how frequently does this occur?	Never; Once a week or less; More than once a week; Every day
Q11	How would you describe the interactions/relationships of your dog with other dogs in your household? (Select all that apply):	They sleep together; They play together; They are tolerant of each other, but not playful; They are mostly tolerant, but occasionally fight (e.g., over food, toys, attention from owners); The “study” dog is fearful of other dogs in the home; They are aggressive with each other (regular fighting, dogs may be kept separated); Not applicable; Other (Please specify)
Q12	Some dogs display aggressive behaviour from time to time. Typical signs of moderate aggression in dogs include barking, growling, and baring teeth. More serious aggression generally includes snapping, lunging, biting, or attempting to bite. By writing in the appropriate number from the scale, please indicate your own dog’s recent tendency to display aggressive behaviour in each of the following contexts: 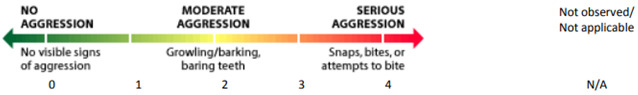	
a	Towards another (familiar) dog in your household.	0–4, N/A
b	When approached at a favorite resting/sleeping place by another (familiar) household dog.	0–4, N/A
c	When approached while eating by another (familiar) household dog.	0–4, N/A
d	When approached while playing with/chewing a favorite toy, bone, object, etc., by another (familiar) household dog.	0–4, N/A
Q13	In question 12, when rating your dog’s behaviour towards another dog in your household on a scale from 0 to 4, which of these statements best describes the “other” dog you were thinking of in that situation (Select Multiple):	Another dog that you own; A dog that you previously owned who lived with your current dog; A friend or family member’s dog who spends time with IN your home; A friend or family member’s dog who you spend time with AWAY from your home; Other (Please Specify)
Q14	Is there anything else you would like to say about your dog’s interactions with other dogs?	Text response

**Table 2:** Mean (±standard error of the mean), median, and range of familiar dog aggression (FDA) scores for dogs who participated in the original cohort (*n* = 235) and the subsample of dogs (*n* = 71/235) who were scored in the follow-up study. FDA scores for dogs reported in the follow-up study are presented for both years (2021, 2022). The total number of dogs in each cohort is presented, with the number who received an FDA score in each cohort displayed in brackets.

FDA Scores	*n*	Mean ± SEM	Median	Range
Original Cohort (2021)	235	0.373 ± 0.05	0	0–4
	(170)			
Follow-up Cohort (2021 scores)	71	0.343 ± 0.08	0	0–2
	(46)			
Follow-up Cohort (2022 scores)	71	0.782 ± 0.11	0.5	0–2.75
	(57)			

**Table 3:** Chi-square tests of association with Fisher’s exact test for study group, and participation of dogs in different socialization contexts. Percentages displayed refer to the proportion of dogs within each group. (Group 1—Singleton dogs with unexpected FDA scores; Group 2—Singleton dogs without FDA scores; Group 3—Multi-home dogs with FDA scores).

**Question/Frequency**	**Total (*n* = 71)**	**Group 1 (*n* = 25)**	**Group 2 (*n* = 25)**	**Group 3 (*n* = 21)**	**Statistic**
	*n* %	*n* %	*n* %	*n* %	χ^2^ *p*
*In-Home Socialization*					14.0 0.026
Never	16 22.5	3 12.0	9 36.0	4 19.1	
Once/week or less	43 60.5	13 52.0	15 60.0	16 76.2	
More than once/week	10 14.1	8 32.0	1 4.0	1 4.7	
Every day	2 2.8	1 4.0	0 0	1 4.7	
*Out-of-Home Socialization*					12.6 0.041
Never	14 19.7	1 4.0	7 28.0	6 28.6	
Once/week or less	48 67.6	17 68.0	17 68.0	14 66.7	
More than once/week	7 9.9	5 20.0	1 4.0	2 9.5	
Every day	2 2.8	2 8.0	0 0	0 0	

**Table 4:** Guardian responses to Question 13 of the follow-up questionnaire, referring to their specific interpretation of “familiar dog” C-BARQ questions (*n* = 71).

**Q13. “When rating your dog’s behaviour towards another dog in your household on a scale from 0–4, which of these statements best describes the ’other’ dog you were thinking of in that situation”**	***n***
Another dog that you own	21
A dog that you previously owned who lived with your current dog	4
A friend or family member’s dog who spends time with your dog IN your home	18
A friend or family member’s dog who you spend time with AWAY from your home	13
No Response	8
Other (Please Specify) *	7

* Other responses: Previous roommate’s dog (*n* = 1); A dog we met regularly (*n* = 1); Only 1 dog in house, others do not visit (*n* = 1); N/A (*n* = 4).

**Table 5:** Quantification of peer-reviewed research papers identified during the scoping review that used the full, mini, or modified version of C-BARQ, and the number of times that familiar dog aggression (FDA) was both reported and found to be significant. References are provided in the footnote for studies that reported any significant FDA findings.

C-BARQ Version	FDA Reported	FDA Findings Significant
Full (*n* = 63)	37/63	18/37 ^1^
Mini (42Q) (*n* = 11)	7/11	4/7 ^2^
Modified/Unreported (*n* = 11)	1/11	1/1 ^3^

^1^ References (Full): [6,7,8,9,10,11,12,13,14,17,30,31,32,33,34,35,36,37]; ^2^ (Mini): [5,29,38,39]; ^3^ (Modified): [40].

## Data Availability

Study 1 data are contained in Supplementary Materials S4.

## Supplementary Materials

The following supporting information can be downloaded at: https://www.mdpi.com/article/10.3390/ani15192876/s1, S1: Diet and Lifestyle Questionnaire (Study 1); S2: FDA Follow-up Questionnaire; S3: List of Final Articles (85) included in Scoping Review; S4: Study 1 Data (.xls file).

## Abbreviations

The following abbreviations are used in this manuscript:

C-BARQ	Canine Behavioral Assessment and Research Questionnaire
FDA	Familiar Dog Aggression
DDF	Dog-Directed Fear
SDF	Stranger-Directed Fear
NSF	Non-Social Fear
PRISMA-ScR	Preferred Reporting Items for Systematic Reviews and Meta-Analyses Scoping Review
