# Supplementary material for: Dog Guardian Interpretation of Familiar Dog Aggression Questions in the C-BARQ: Do We Need to Redefine “Familiar”?
Source: Animals (Basel). 2025 Sep 30;15(19):2876. doi: 10.3390/ani15192876 (PMC12524257; doi:10.3390/ani15192876)
Supplement: Supplementary file 1 [file animals-15-02876-s001.zip › Suppl_S3_ScR Refs_Animals.docx]

**Supplementary Materials S3: References included in Scoping Review**

Barnard, S., Flint, H., Diana, A., Shreyer, T., Arrazola, A., Serpell, J., Croney, C., 2023. Management and behavioral factors associated with rehoming outcomes of dogs retired from commercial breeding kennels. *PLoS ONE* **18**, e0282459. <https://doi.org/10.1371/journal.pone.0282459>

Barnard, S., Siracusa, C., Reisner, I., Valsecchi, P., Serpell, J.A., 2012. Validity of model devices used to assess canine temperament in behavioral tests. *Applied Animal Behaviour Science* **138**, 79–87. <https://doi.org/10.1016/j.applanim.2012.02.017>

Barnard, S., Wells, D.L., Milligan, A.D.S., Arnott, G., Hepper, P.G., 2018. Personality traits affecting judgement bias task performance in dogs (*Canis familiaris*). *Scientific Reports* **8**, 6660. <https://doi.org/10.1038/s41598-018-25224-y>

Bray, E.E., Levy, K.M., Kennedy, B.S., Duffy, D.L., Serpell, J.A., MacLean, E.L., 2019. Predictive models of assistance dog training outcomes using the canine behavioral assessment and research questionnaire and a standardized temperament evaluation. *Frontiers in Veterinary Science* **6**, 49. <https://doi.org/10.3389/fvets.2019.00049>

Bray, E.E., Sammel, M.D., Seyfarth, R.M., Serpell, J.A., Cheney, D.L., 2017. Temperament and problem solving in a population of adolescent guide dogs. *Animal Cognition* **20**, 923–939. <https://doi.org/10.1007/s10071-017-1112-8>

Buttner, A.P., Awalt, S.L., Strasser, R., 2023. Early life adversity in dogs produces altered physiological and behavioral responses during a social stress‐buffering paradigm. *Journal of the Experimental Analysis of Behavior* **120**, 6–20. <https://doi.org/10.1002/jeab.856>

Chen, Q., Xu, Y., Christiaen, E., Wu, G.-R., De Witte, S., Vanhove, C., Saunders, J., Peremans, K., Baeken, C., 2023. Structural connectome alterations in anxious dogs: a DTI-based study. *Scientific Reports* **13**, 9946. <https://doi.org/10.1038/s41598-023-37121-0>

Clay, L., Paterson, M.B.A., Bennett, P., Perry, G., Phillips, C.C.J., 2020. Comparison of canine behaviour scored using a shelter behaviour assessment and an owner completed questionnaire, C-BARQ. *Animals* **10**(10), 1797. <https://doi.org/10.3390/ani10101797>

Cook, P.F., Spivak, M., Berns, G.S., 2014. One pair of hands is not like another: Caudate BOLD response in dogs depends on signal source and canine temperament. *PeerJ* **2**, e596. <https://doi.org/10.7717/peerj.596>

Dalal, S., Hall, N.J., 2019. Behavioral persistence is associated with poorer olfactory discrimination learning in domestic dogs. *Behavioural Processes* **162**, 64–71. <https://doi.org/10.1016/j.beproc.2019.01.010>

De Meester, R.H., De Bacquer, D., Peremans, K., Vermeire, S., Planta, D.J., Coopman, F., Audenaert, K., 2008. A preliminary study on the use of the Socially Acceptable Behavior Test as a test for shyness/confidence in the temperament of dogs. *Journal of Veterinary Behavior: Clinical Applications and Research* **3**, 161–170. <https://doi.org/10.1016/j.jveb.2007.10.005>

Doane, M., Sarenbo, S., 2019. A modified combined C-BARQ and QoL for both the companion dog and its owner: An embryo to a companion dog welfare assessment? *Applied Animal Behaviour Science* **213**, 91–106. <https://doi.org/10.1016/j.applanim.2019.02.012>

Dockx, R., Baeken, C., De Bundel, D., Saunders, J., Van Eeckhaut, A., Peremans, K., 2019. Accelerated high-frequency repetitive transcranial magnetic stimulation positively influences the behavior, monoaminergic system, and cerebral perfusion in anxious aggressive dogs: A case study. *Journal of Veterinary Behavior* **33**, 108–113. <https://doi.org/10.1016/j.jveb.2019.07.004>

Dodman, N.H., Brown, D.C., Serpell, J.A., 2018. Associations between owner personality and psychological status and the prevalence of canine behavior problems. *PLoS ONE* **13**(2), e0192846. <https://doi.org/10.1371/journal.pone.0192846>

Duffy, D.L., Hsu, Y., Serpell, J.A., 2008. Breed differences in canine aggression. *Applied Animal Behaviour Science* **114**, 441–460. <https://doi.org/10.1016/j.applanim.2008.04.006>

Duffy, D.L., Kruger, K.A., Serpell, J.A., 2014. Evaluation of a behavioral assessment tool for dogs relinquished to shelters. *Preventive Veterinary Medicine* **117**, 601–609. <https://doi.org/10.1016/j.prevetmed.2014.10.003>

Duffy, D.L., Serpell, J.A., 2012. Predictive validity of a method for evaluating temperament in young guide and service dogs. *Applied Animal Behaviour Science* **138**, 99–109. <https://doi.org/10.1016/j.applanim.2012.02.011>

Edwards, P.T., Hazel, S.J., Browne, M., Serpell, J.A., McArthur, M.L., Smith, B.P., 2019. Investigating risk factors that predict a dog’s fear during veterinary consultations. *PLoS ONE* **14**, e0215416. <https://doi.org/10.1371/journal.pone.0215416>

Farhoody, P., Mallawaarachchi, I., Tarwater, P.M., Serpell, J.A., Duffy, D.L., Zink, C., 2018. Aggression toward familiar people, strangers, and conspecifics in gonadectomized and intact dogs. *Frontiers in Veterinary Science* **5**, 18. <https://doi.org/10.3389/fvets.2018.00018>

Farmer-Dougan, V., Quick, A., Harper, K., Schmidt, K., Campbell, D., 2014. Behavior of hearing or vision impaired and normal hearing and vision dogs (*Canis lupis familiaris*): Not the same, but not that different. *Journal Of Veterinary Behavior-Clinical Applications And Research* **9**, 316–323.<https://doi.org/10.1016/j.jveb.2014.07.002>

Flint, H.E., Coe, J.B., Pearl, D.L., Serpell, J.A., Niel, L., 2018. Effect of training for dog fear identification on dog owner ratings of fear in familiar and unfamiliar dogs. *Applied Animal Behaviour Science* **208**, 66–74. <https://doi.org/10.1016/j.applanim.2018.08.002>

Flint, H.E., Coe, J.B., Serpell, J.A., Pearl, D.L., Niel, L., 2017. Risk factors associated with stranger-directed aggression in domestic dogs. *Applied Animal Behaviour Science* **197**, 45–54. <https://doi.org/10.1016/j.applanim.2017.08.007>

Friedrich, J., Arvelius, P., Strandberg, E., Polgar, Z., Wiener, P., Haskell, M.J., 2019a. The interaction between behavioural traits and demographic and management factors in German Shepherd dogs. *Applied Animal Behaviour Science* **211**, 67–76. <https://doi.org/10.1016/j.applanim.2018.12.004>

Friedrich, J., Strandberg, E., Arvelius, P., Sánchez-Molano, E., Pong-Wong, R., Hickey, J.M., Haskell, M.J., Wiener, P., 2019b. Genetic dissection of complex behaviour traits in German Shepherd dogs. *Heredity* **123**, 746–758. <https://doi.org/10.1038/s41437-019-0275-2>

Ghirlanda, S., Acerbi, A., Herzog, H., Serpell, J.A., 2013. Fashion vs function in cultural evolution: The case of dog breed popularity. *PLoS ONE* **8**(9), e74770. <https://doi.org/10.1371/journal.pone.0074770>

Grigg, E.K., Nibblett, B.M., Sacks, B.N., Hack, R., Serpell, J.A., Hart, L., 2016. Genetic and behavioral characteristics of the St Kitts ‘island dog.’ *Applied Animal Behaviour Science* **178**, 88–95. <https://doi.org/10.1016/j.applanim.2016.02.002>

Hare, E., Joffe, E., Wilson, C., Serpell, J., Otto, C.M., 2021a. Behavior traits associated with career outcome in a prison puppy-raising program. *Applied Animal Behaviour Science* **236**, 105218. <https://doi.org/10.1016/j.applanim.2021.105218>

Hare, E., Kelsey, K.M., Niedermeyer, G.M., Otto, C.M., 2021b. Long-term behavioral resilience in search-and-rescue dogs responding to the September 11, 2001 terrorist attacks. *Applied Animal Behaviour Science* **234**, 105173. <https://doi.org/10.1016/j.applanim.2020.105173>

Hare, E., Kelsey, K.M., Serpell, J.A., Otto, C.M., 2018. Behavior differences between search-and-rescue and pet dogs. *Frontiers in Veterinary Science* **5**, 118. <https://doi.org/10.3389/fvets.2018.00118>

Harvey, N., Craigon, P., Blythe, S., England, G., Asher, L., 2016. Social rearing environment influences dog behavioral development. *Journal Of Veterinary Behavior-Clinical Applications And Research* **16**, 13–21. <https://doi.org/10.1016/j.jveb.2016.03.004>

Hecht, E.E., Zapata, I., Alvarez, C.E., Gutman, D.A., Preuss, T.M., Kent, M., Serpell, J.A., 2021. Neurodevelopmental scaling is a major driver of brain-behavior differences in temperament across dog breeds. *Brain Structure & Function* **226**, 2725–2739. <https://doi.org/10.1007/s00429-021-02368-8>

Heys, M., Lloyd, I., Westgarth, C., 2023. “Bowls are boring”: Investigating enrichment feeding for pet dogs and the perceived benefits and challenges. *The Veterinary Record* **194**(4), e3169. <https://doi.org/10.1002/vetr.3169>

Hoffman, C.L., Chen, P., Serpell, J.A., Jacobson, K.C., 2013. Do dog behavioral characteristics predict the quality of the relationship between dogs and their owners? *Human-Animal Interaction Bulletin* **1**, 20–37

Hoffman, C.L., Suchak, M., 2017. Dog rivalry impacts following behavior in a decision-making task involving food. *Animal Cognition* **20**, 689–701. <https://doi.org/10.1007/s10071-017-1091-9>

Hsu Y, Serpell J.A., 2003. Development and validation of a questionnaire for measuring behavior and temperament traits in pet dogs. *Journal of the American Veterinary Medical Association* **223**, 1293–1300. <https://doi.org/10.2460/javma.2003.223.1293>

Hunt, R.L., England, G.C.W., Asher, L., Whiteside, H., Harvey, N.D., 2020. Concurrent and predictive criterion validity of a puppy behaviour questionnaire for predicting training outcome in juvenile guide dogs. *Animals***10**(12), 2382. <https://doi.org/10.3390/ani10122382>

Ilska J., Haskell M.J., Blott S.C., Sánchez-Molano E., Polgar Z., Lofgren S.E., Clements D.N., Wiener P., 2017. Genetic characterization of dog personality traits. *Genetics* **206**, 1101–1111. <https://doi.org/10.1534/genetics.116.192674>

Lazarowski, L., Rogers, B., Krichbaum, S., Haney, P., Smith, J.G., Waggoner, P., 2021. Validation of a behavior test for predicting puppies’ suitability as detection dogs. *Animals***11**(4), 993. <https://doi.org/10.3390/ani11040993>

Levitin, H., Hague, D.W., Ballantyne, K.C., Selmic, L.E., 2019. Behavioral changes in dogs with idiopathic epilepsy compared to other medical populations. *Frontiers in Veterinary Science* **6**, 396. <https://doi.org/10.3389/fvets.2019.00396>

Lofgren, S.E., Wiener, P., Blott, S.C., Sanchez-Molano, E., Woolliams, J.A., Clements, D.N., Haskell, M.J., 2014. Management and personality in Labrador Retriever dogs. *Applied Animal Behaviour Science* **156**, 44–53. <https://doi.org/10.1016/j.applanim.2014.04.006>

Lopresti-Goodman, S., Bensmiller, N., 2022. Former laboratory dogs’ psychological and behavioural characteristics. *Veterinarni Medicina* **67**, 599–610. <https://doi.org/10.17221/139/2021-VETMED>

MacLean, E.L., Snyder-Mackler, N., VonHoldt, B.M., Serpell, J.A., 2019. Highly heritable and functionally relevant breed differences in dog behaviour. *Proceedings of the Royal Society B: Biological Sciences* **286**, 20190716. <https://doi.org/10.1098/rspb.2019.0716>

McAuliffe, L.R., Koch, C.S., Serpell, J., Campbell, K.L., 2022. Associations between atopic dermatitis and anxiety, aggression, and fear-based behaviors in dogs. *Journal of the American Animal Hospital Association* **58**, 161–167. <https://doi.org/10.5326/JAAHA-MS-7210>

McCullough, A., Jenkins, M.A., Ruehrdanz, A., Gilmer, M.J., Olson, J., Pawar, A., Holley, L., Sierra-Rivera, S., Linder, D.E., Pichette, D., Grossman, N.J., Hellman, C., Guérin, N.A., O’Haire, M.E., 2018. Physiological and behavioral effects of animal-assisted interventions on therapy dogs in pediatric oncology settings. *Applied Animal Behaviour Science* **200**, 86–95. <https://doi.org/10.1016/j.applanim.2017.11.014>

McGreevy, P.D., Georgevsky, D., Carrasco, J., Valenzuela, M., Duffy, D.L., Serpell, J.A., 2013. Dog behavior co-varies with height, bodyweight and skull shape. *PLoS ONE* **8**, e80529. <https://doi.org/10.1371/journal.pone.0080529>

McGreevy, P.D., Wilson, B., Starling, M.J., Serpell, J.A., 2018. Behavioural risks in male dogs with minimal lifetime exposure to gonadal hormones may complicate population-control benefits of desexing. *PLoS ONE* **13**, e0196284. <https://doi.org/10.1371/journal.pone.0196284>

McMillan, F., Serpell, J., Duffy, D., Masaoud, E., Dohoo, I., 2013. Differences in behavioral characteristics between dogs obtained as puppies from pet stores and those obtained from noncommercial breeders. *Journal of the American Veterinary Medical Association* **242**, 1359–1363. <https://doi.org/10.2460/javma.242.10.1359>

McMillan, F.D., Duffy, D.L., Serpell, J.A., 2011. Mental health of dogs formerly used as ‘breeding stock’ in commercial breeding establishments. *Applied Animal Behaviour Science* **135**, 86–94. <https://doi.org/10.1016/j.applanim.2011.09.006>

McMillan, F.D., Duffy, D.L., Zawistowski, S.L., Serpell, J.A., 2015. Behavioral and psychological characteristics of canine victims of abuse. *Journal of Applied Animal Welfare Science* **18**, 92–111. <https://doi.org/10.1080/10888705.2014.962230>

McMillan, F.D., Vanderstichel, R., Stryhn, H., Yu, J., Serpell, J.A., 2016. Behavioural characteristics of dogs removed from hoarding situations. *Applied Animal Behaviour Science* **178**, 69–79. <https://doi.org/10.1016/j.applanim.2016.02.006>

Moxon, R., Freeman, S., Payne, R., Corr, S., England, G.C.W., 2022. A prospective cohort study investigating the behavioural development of bitches in a guide dog training programme neutered prepubertally or post-pubertally. *Frontiers in Veterinary Science* **9**, 902775. <https://doi.org/10.3389/fvets.2022.902775>

Nagasawa, M., Kanbayashi, S., Mogi, K., Serpell, J., Kikusui, T., 2016. Comparison of behavioral characteristics of dogs in the United States and Japan. *Journal of Veterinary Medical Science* **78**, 231–238. <https://doi.org/10.1292/jvms.15-0253>

Packer, R.M.A., De Risio, L., Volk, H.A., 2017. Investigating the potential of the anti-epileptic drug imepitoin as a treatment for co-morbid anxiety in dogs with idiopathic epilepsy. *BMC Veterinary Research* **13**, 90. <https://doi.org/10.1186/s12917-017-1000-0>

Plueckhahn, T.C., Schneider, L.A., Delfabbro, P.H., 2022. Comparing owner-rated dog temperament measures and a measure of owner personality: An exploratory study. *Anthrozoös* **36**(1), 53-67. <https://doi.org/10.1080/08927936.2022.2062870>

Powell, L., Duffy, D.L., Kruger, K.A., Watson, B., Serpell, J.A., 2021a. Relinquishing owners underestimate their dog’s behavioral problems: deception or lack of knowledge? *Frontiers in Veterinary Science* **8**, 734973. <https://doi.org/10.3389/fvets.2021.734973>

Powell, L., Lee, B., Reinhard, C., Morris, M., Satriale, D., Serpell, J., Watson, B., 2022. Returning a shelter dog: the role of owner expectations and dog behavior. *Animals* **12**(9), 1053. <https://doi.org/10.3390/ani12091053>

Powell, L., Stefanovski, D., Siracusa, C., Serpell, J., 2021b. owner personality, owner-dog attachment, and canine demographics influence treatment outcomes in canine behavioral medicine cases. *Frontiers in Veterinary Science* **7**, 630931. <https://doi.org/10.3389/fvets.2020.630931>

Rayment, D.J., Peters, R.A., Marston, L.C., De Groef, B., 2020. Relationships between serum serotonin, plasma cortisol, and behavioral factors in a mixed-breed, sex, and age group of pet dogs. *Journal of Veterinary Behavior* **38**, 96–102. <https://doi.org/10.1016/j.jveb.2020.05.007>

Rayment, D.J., Peters, R.A., Marston, L.C., De Groef, B., 2016. Investigating canine personality structure using owner questionnaires measuring pet dog behaviour and personality. *Applied Animal Behaviour Science* **180**, 100–106. <https://doi.org/10.1016/j.applanim.2016.04.002>

Riemer, S., Thompson, H., Burman, O.H.P., 2018. Behavioural responses to unexpected changes in reward quality. *Scientific Reports* **8**, 16652. <https://doi.org/10.1038/s41598-018-35056-5>

Roth, L.S.V., Faresjö, Å., Theodorsson, E., Jensen, P., 2016. Hair cortisol varies with season and lifestyle and relates to human interactions in German shepherd dogs. *Scientific Reports* **6**, 19631. <https://doi.org/10.1038/srep19631>

Sanders, A.R., Bhongir, N., vonHoldt, B., Pellegrini, M., 2022. Association of DNA methylation with energy and fear-related behaviors in canines. *Frontiers in Psychology* **13**, 1025494. <https://doi.org/10.3389/fpsyg.2022.1025494>

Schneider, L.A., Delfabbro, P.H., Burns, N.R., 2013. Temperament and lateralization in the domestic dog (*Canis familiaris*). *Journal of Veterinary Behavior* **8**, 124–134. <https://doi.org/10.1016/j.jveb.2012.06.004>

Segurson S.A., Serpell J.A., Hart B.L., 2005. Evaluation of a behavioral assessment questionnaire for use in the characterization of behavioral problems of dogs relinquished to animal shelters. *Journal of the American Veterinary Medical Association* **227**, 1755–61. <https://doi.org/10.2460/javma.2005.227.1755>

Serpell, J.A., Duffy, D.L., 2016. Aspects of juvenile and adolescent environment predict aggression and fear in 12-month-old guide dogs. *Frontiers in Veterinary Science* **3**, 49. <https://doi.org/10.3389/fvets.2016.00049>

Serpell, J.A., Hsu, Y., 2005. Effects of breed, sex, and neuter status on trainability in dogs. *Anthrozoös* **18**, 196–207. <https://doi.org/10.2752/089279305785594135>

Shouldice, V.L., Edwards, A.M., Serpell, J.A., Niel, L., Robinson, J.A.B., 2019. Expression of behavioural traits in goldendoodles and labradoodles. *Animals* **9**(12), 1162. <https://doi.org/10.3390/ani9121162>

Showalter, R., Polewan, R., Drury, S., 2018. The influence of sex and temperament on spatial learning in domestic dogs. *International Journal of Humanities and Social Science* **8**(6). <http://doi.org/10.30845/ijhss.v8n6a1>

Smith, B.P., Browne, M., Serpell, J.A., 2017. Owner-reported behavioural characteristics of dingoes (*Canis dingo*) living as companion animals: A comparison to ‘modern’ and ‘ancient’ dog breeds. *Applied Animal Behaviour Science* **187**, 77–84. <https://doi.org/10.1016/j.applanim.2016.11.010>

Starling, M., Fawcett, A., Wilson, B., Serpell, J., McGreevy, P., 2019. Behavioural risks in female dogs with minimal lifetime exposure to gonadal hormones. *PLoS ONE* **14**, e0223709. <https://doi.org/10.1371/journal.pone.0223709>

Stellato, A.C., Flint, H.E., Widowski, T.M., Serpell, J.A., Niel, L., 2017. Assessment of fear-related behaviours displayed by companion dogs (*Canis familiaris*) in response to social and non-social stimuli. *Applied Animal Behaviour Science* **188**, 84–90. <https://doi.org/10.1016/j.applanim.2016.12.007>

Sumridge, M.H., Suchak, M., Hoffman, C.L., 2021. Owner-reported attachment and behavior characteristics of new guinea singing dogs living as companion animals. *Anthrozoös* **34**, 375-392. <https://doi.org/10.1080/08927936.2021.1898218>

Tandon, D., Ressler, K., Petticord, D., Papa, A., Jiranek, J., Wilkinson, R., Kartzinel, R.Y., Ostrander, E.A., Burney, N., Borden, C., Udell, M.A.R., VonHoldt, B.M., 2019. Homozygosity for mobile element insertions associated with wbscr17 could predict success in assistance dog training programs. *Genes* **10**. <https://doi.org/10.3390/genes10060439>

Thielke, L.E., Udell, M.A.R., 2019. Evaluating cognitive and behavioral outcomes in conjunction with the secure base effect for dogs in shelter and foster environments. *Animals***9**(11), 932. <https://doi.org/10.3390/ani9110932>

van den Berg, S.M., Heuven, H.C.M., van den Berg, L., Duffy, D.L., Serpell, J.A., 2010. Evaluation of the C-BARQ as a measure of stranger-directed aggression in three common dog breeds. *Applied Animal Behaviour Science* **124**, 136–141. <https://doi.org/10.1016/j.applanim.2010.02.005>

van Rooy, D., Thomson, P.C., McGreevy, P.D., Wade, C.M., 2018. Risk factors of separation-related behaviours in Australian retrievers. *Applied Animal Behaviour Science* **209**, 71–77. <https://doi.org/10.1016/j.applanim.2018.09.001>

von Rentzell, K.A., van Haaften, K., Morris, A., Protopopova, A., 2022. Investigation into owner-reported differences between dogs born in versus imported into Canada. *PLoS ONE* **17**, e0268885. <https://doi.org/10.1371/journal.pone.0268885>

Watanangura, A., Meller, S., Suchodolski, J.S., Pilla, R., Khattab, M.R., Loderstedt, S., Becker, L.F., Bathen-Nöthen, A., Mazzuoli-Weber, G., Volk, H.A., 2022. The effect of phenobarbital treatment on behavioral comorbidities and on the composition and function of the fecal microbiome in dogs with idiopathic epilepsy. *Frontiers in Veterinary Science* **9**, 933905. <https://doi.org/10.3389/fvets.2022.933905>

Wauthier, L.M., Williams, J.M., 2018. Using the mini C-BARQ to investigate the effects of puppy farming on dog behaviour. *Applied Animal Behaviour Science* **206**, 75–86. <https://doi.org/10.1016/j.applanim.2018.05.024>

Wells, D.L., Hepper, P.G., Milligan, A.D.S., Barnard, S., 2019. Lack of association between paw preference and behaviour problems in the domestic dog, *Canis familiaris*. *Applied Animal Behaviour Science* **210**, 81–87. <https://doi.org/10.1016/j.applanim.2018.10.008>

Wilson, B., Serpell, J., Herzog, H., McGreevy, P., 2018. Prevailing clusters of canine behavioural traits in historical us demand for dog breeds (1926^-^2005). *Animals* **8**(11), 197. <https://doi.org/10.3390/ani8110197>

Zapata, I., Eyre, A., Alvarez, C., 2023. Psychological stress is associated with increased cancer risk in dogs. *Animals* **13**(11), 1869. <https://doi.org/10.3390/ani13111869>

Zapata, I., Eyre, A.W., Alvarez, C.E., Serpell, J.A., 2022a. Latent class analysis of behavior across dog breeds reveal underlying temperament profiles. *Scientific Reports* **12**, 15627. <https://doi.org/10.1038/s41598-022-20053-6>

Zapata, I., Lilly, M.L., Herron, M.E., Serpell, J.A., Alvarez, C.E., 2022b. Genetic testing of dogs predicts problem behaviors in clinical and nonclinical samples. *BMC Genomics* **23**, 102. <https://doi.org/10.1186/s12864-022-08351-9>

Zapata, I., Serpell, J.A., Alvarez, C.E., 2016. Genetic mapping of canine fear and aggression. *BMC Genomics* **17**, 572. <https://doi.org/10.1186/s12864-016-2936-3>
