# Supplementary material for: Dog Guardian Interpretation of Familiar Dog Aggression Questions in the C-BARQ: Do We Need to Redefine “Familiar”?
Source: Animals (Basel). 2025 Sep 30;15(19):2876. doi: 10.3390/ani15192876 (PMC12524257; doi:10.3390/ani15192876)
Supplement: Supplementary file 1 [file animals-15-02876-s001.zip › Suppl_S1_D&L Questionnaire_Animals.docx]

**Supplementary Materials S1: Diet & Lifestyle Questionnaire**

**Lifestyle & Routine**

**How many dogs live in your home?**

1

2

3

4+

**What would you consider to be your dog’s main source of exercise:**

Walking (ie neighbourhood walks)

Running

Hiking

Fetch/Frisbee

Garden/Yard

Doggie Daycare

None/Not Applicable

**On average, how frequently does your dog exercise away from your property (eg. Walks, hiking etc) each week?**

Never

Less than once per week

Once per week

2-4 times per week

Daily

**On average, how much time does your dog spend exercising away from your property (eg. Walks, hiking etc) each week?**

Less than one hour per week

1-2 hours per week

2-4 hours per week

4-6 hours per week

6+ hours per week

**How frequently does your dog encounter people outside your family?**

Never

Less than once per week

Once per week

2-4 times per week

Daily

**How frequently does your dog encounter dogs outside your family?**

Never

Less than once per week

Once per week

2-4 times per week

Daily

**Diet**

**Does your dog have scheduled meal times, or are they free-fed?**

Scheduled meals

Free-fed

**If your dog has scheduled meal times, how many times per day does your dog eat?**

One

Two

Three

Four or more

**How long has your dog been fed on this schedule?**

Less than a month

1-3 months

3 – 6 months

6+ months

**What type of food forms the majority of your dog’s diet? (select all that apply)**

Kibble

Canned/Wet food

Raw

Home-cooked

**What brand of food do you feed your dog? (eg. Purina, Acana, Orijen, Royal Canin)**

____________________________________

**What formula of food do you feed your dog? (eg. Kirkland Salmon & Sweet Potato, Acana Wild Coast, Go! Sensitivity Salmon Formula) Please provide as much information as possible so we can search the ingredients and nutritional information – you can also email a photo of the food packaging to** [**sdp575@mun.ca**](mailto:sdp575@mun.ca)

_______________________________________

**How long has your dog been eating this food?**

Less than a month

1-3 months

3 – 6 months

6+ months

**Do you supplement your dog’s diet regularly (ie. more than once per week) with any of the following foods (select all that apply):**

Green vegetables (broccoli, spinach, kale, peas, beans)

Salad vegetables (tomato, peppers, cucumber)

Fruits (blueberries, banana, strawberries, apple)

Poultry

Red meats

Fish

Deli meats (hot dogs, ham, bologna)

Starchy vegetables (potato, sweet potato)

Yogurt

Peanut butter

From the following graphic, how would you rate your dog’s body condition?


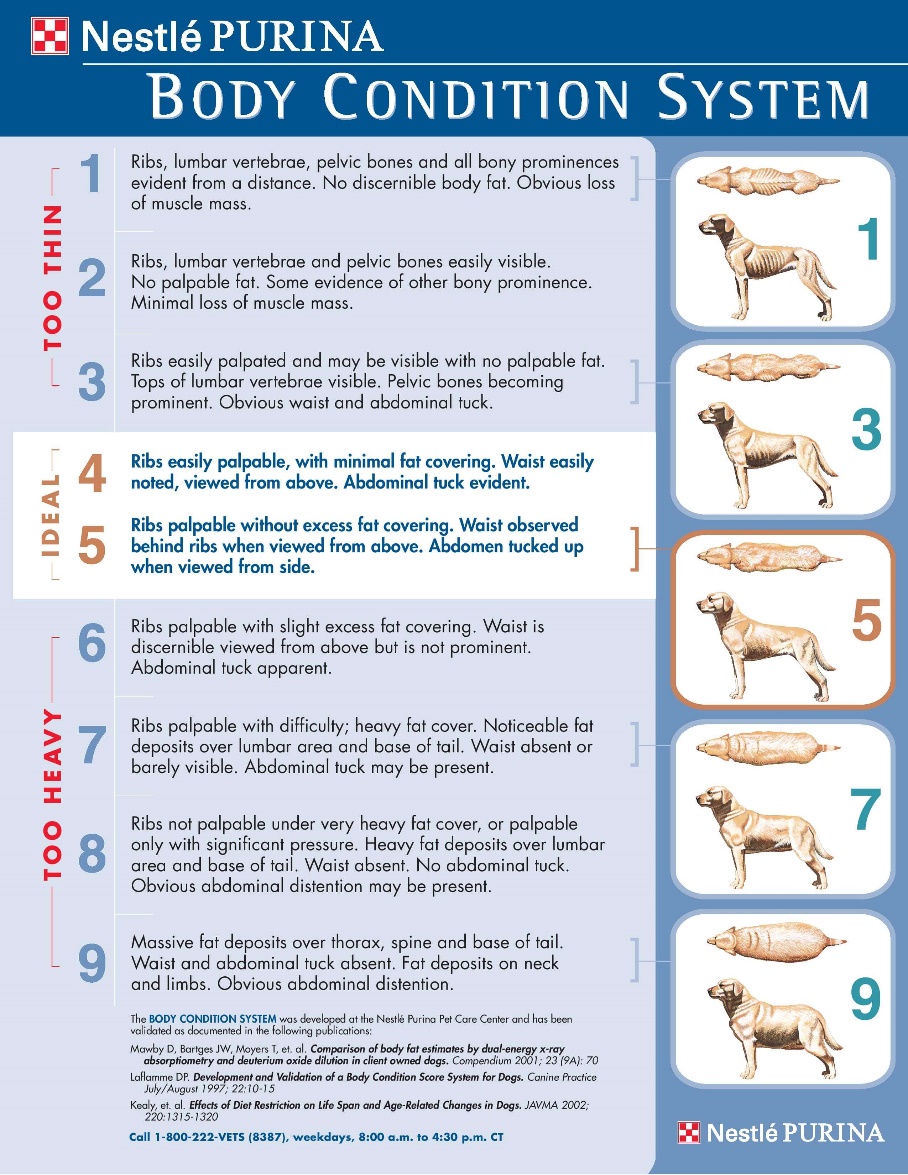
1

3

5

7

9

**Medical**

**Does your dog currently eat a veterinary-prescribed diet?**

Yes

No

**If yes, please specify the brand, formula and reason for the particular diet (eg. Purina ProPlan Neurocare kibble, for epilepsy):**

____________________________________________

**Has your dog ever taken a dewormer, such as Sentinel or Strongid?**

Yes

No

**On average, how frequently does your dog take a dewormer?**

Every month

Every 3 months

Every 6 months

Every year

Never

**When did your dog last receive their dewormer?**

_____________________________________

**Has your dog ever taken flea medication, such as Advantage Multi?**

Yes

No

**On average, how frequently does your dog take flea medication?**

Every month

Every 3 months

Every 6 months

Every year

Never

**When did your dog last receive their flea medication?**

_____________________________________

**Has your dog been vaccinated against parvovirus, distemper and parainfluenza? (hint – this is the vaccination your puppy gets at 8, 12 and 16 weeks, then as a booster in adulthood)**

Yes

No

**On average, how frequently has your dog been vaccinated since their puppy boosters?**

Every year

Every 2 years

Every 3 years

Less than every 3 years

Never

**When did your dog last receive their vaccinations for parvovirus, distemper and parainfluenza?**

_____________________________________

**Does your dog currently taking any medication? If so, please specify:**

______________________________________

**Does your dog receive any of the following supplements:**

Omega 3/6 oils

CBD products

Fortiflora/Probiotics

Other: __________________

**On average, how many times does your dog defecate each day?**

One

Two

Three

More than three


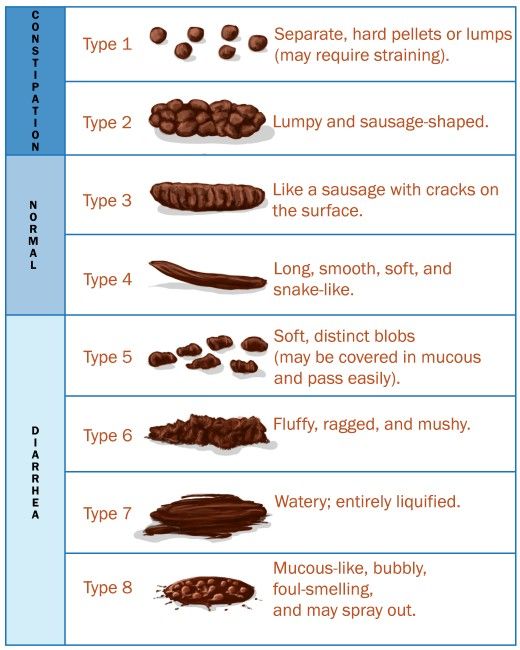
**On average, which of these movements most accurately represent your dog’s bowel movements?**

1

2

3

4

5

6

7

8
