# Supplementary material for: Dog Guardian Interpretation of Familiar Dog Aggression Questions in the C-BARQ: Do We Need to Redefine “Familiar”?
Source: Animals (Basel). 2025 Sep 30;15(19):2876. doi: 10.3390/ani15192876 (PMC12524257; doi:10.3390/ani15192876)
Supplement: Supplementary file 1 [file animals-15-02876-s001.zip › Suppl_S2_FDA Questionnaire_Animals.docx]

**Supplementary Materials S2: FDA Investigation Questionnaire**

Thank you for your continued participation in our study! If you do not wish to answer a particular question, you can leave the space blank (for text responses), or click “N/A” or “Skip” where appropriate. If at any point you decide not to continue with the questionnaire or do not wish to submit your responses, you can close your browser.

Q1: What is your name?

Q2: What is your email address?

Q3: What is your dog’s name?

Q4: What is your dog's breed? (If your dog is a mixed breed, and you can confirm the breeds in your dog's background, either from knowing the parents' breeds, or from genetic testing results, please enter the breed mix, e.g., 'husky-beagle mix'. If you are unsure about the mix, please enter 'mixed breed'.)

Q5: How many dogs currently live in your home? (1, 2, 3, 4+)

Q6: How long has your household had this number of dogs? (Less than 1 month, 1-3 months, 3-6, 6-12, over 12 months)

Q7: If your dog currently lives alone, have they previously lived with another dog? (Yes, No, N/A)

Q8: If yes, which of the following statements best describes the transition from living with another dog to becoming the only dog in the household [Select multiple]:

The other dog passed away;

the other dog belonged to a friend/family member and living arrangements changed;

the other dog was rehomed;

the other dog was a temporary foster;

not applicable;

other

Q9: Do you take your dog to visit other dogs at their home, or have other dogs over to visit at your home? If so, how frequently does this occur? These ‘other dogs’ might belong to neighbours, family or friends.

Never;

Once a week or less;

More than once a week;

Every day

Q10: Do you take your dog to socialize with other dogs away from a household environment (eg. group walks/hikes with other dog owners, dog parks)? If so, how frequently does this occur?

Never;

Once a week or less;

More than once a week;

Every day

Q11: How would you describe the interactions/relationships of your dog with other dogs in your household? (Select all that apply):

They sleep together;

They play together;

They are tolerant of each other, but not playful;

They are mostly tolerant, but occasionally fight (e.g., over food, toys, attention from owners);

The 'study' dog is fearful of other dogs in the home;

They are aggressive with each other (regular fighting, dogs may be kept separated);

Not Applicable;

Other (Please specify))

Q12. Some dogs display aggressive behavior from time to time. Typical signs of moderate aggression in dogs include barking, growling and baring teeth. More serious aggression generally includes snapping, lunging, biting, or attempting to bite. By writing in the appropriate number from the scale, please indicate your own dog's recent tendency to display aggressive behavior in each of the following contexts:


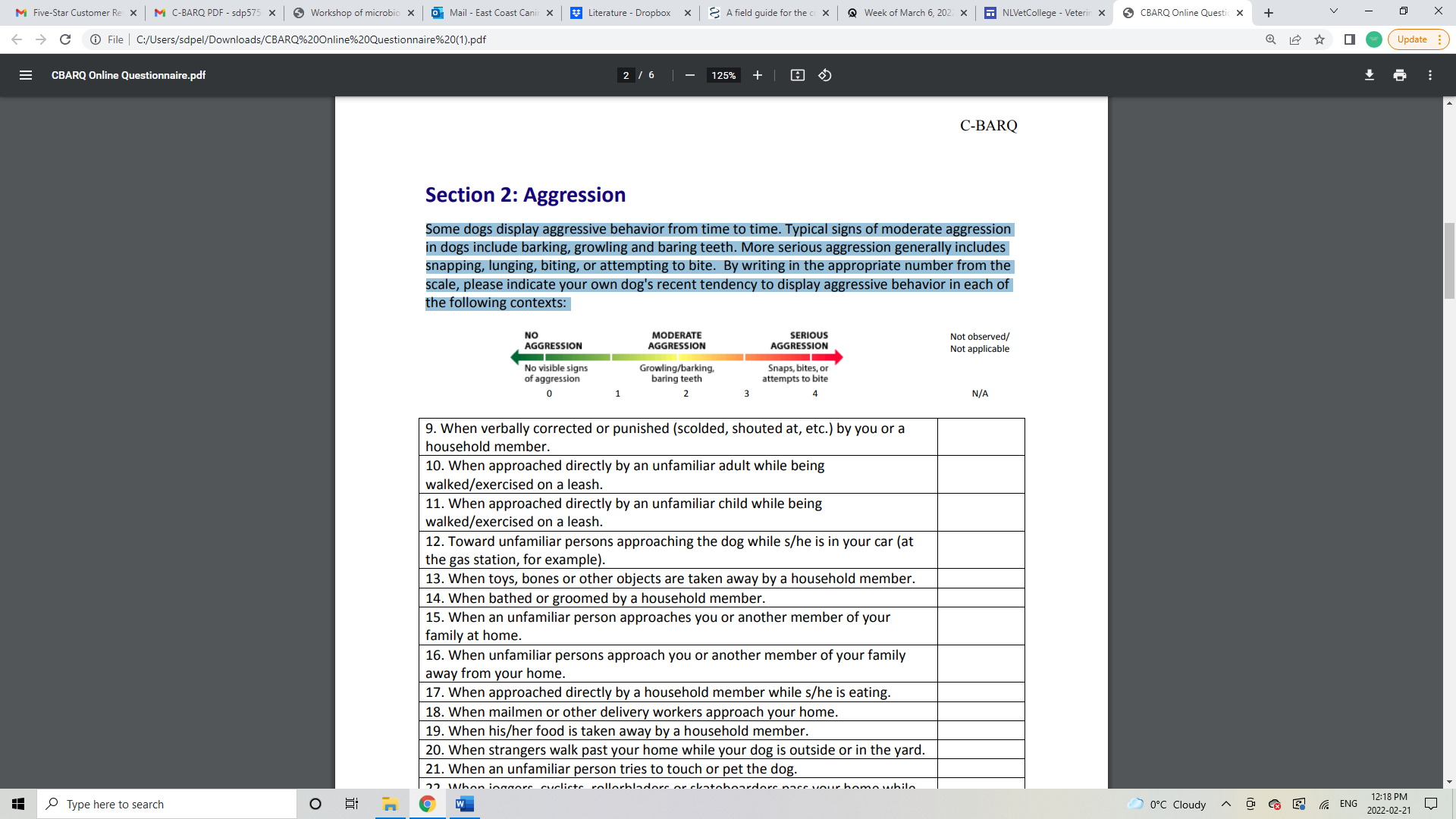


Q12a. Towards another (familiar) dog in your household.

Q12b. When approached at a favorite resting/sleeping place by another (familiar) household dog.

Q12c. When approached while eating by another (familiar) household dog.

Q12d. When approached while playing with/chewing a favorite toy, bone, object, etc., by another (familiar) household dog.

Q13: In question 12, when rating your dog’s behaviour towards another dog in your household on a scale from 0-4, which of these statements best describes the “other” dog you were thinking of in that situation [Select Multiple]:

another dog that you own;

a dog that you have previously owned who lived with your current dog;

a friend or family member’s dog who spends time with IN your home;

a friend or family member’s dog who you spend time with AWAY from your home;

other [Please Specify:])

Q14. Is there anything else you would like to say about your dog’s interactions with other dogs? (TEXT)
